# Hierarchical sequence labeling for extracting BEL statements from biomedical literature

**DOI:** 10.1186/s12911-019-0758-3

**Published:** 2019-04-09

**Authors:** Suwen Liu, Yifan Shao, Longhua Qian, Guodong Zhou

**Affiliations:** School of Computer Science and Technology, Soochow University, Suzhou, China

**Keywords:** Causal relationship extraction, Biological expression language, Hierarchical sequence labeling, Word alignment

## Abstract

**Background:**

Extracting relations between bio-entities from biomedical literature is often a challenging task and also an essential step towards biomedical knowledge expansion. The BioCreative community has organized a shared task to evaluate the robustness of the causal relationship extraction algorithms in Biological Expression Language (BEL) from biomedical literature.

**Method:**

We first map the sentence-level BEL statements in the BC-V training corpus to the corresponding text segments, thus generating hierarchically tagged training instances. A hierarchical sequence labeling model was afterwards induced from these training instances and applied to the test sentences in order to construct the BEL statements.

**Results:**

The experimental results on extracting BEL statements from BioCreative V Track 4 test corpus show that our method achieves promising performance with an overall F-measure of 31.6%. Furthermore, it has the potential to be enhanced by adopting more advanced machine learning approaches.

**Conclusion:**

We propose a framework for hierarchical relation extraction using hierarchical sequence labeling on the instance-level training corpus derived from the original sentence-level corpus via word alignment. Its main advantage is that we can make full use of the original training corpus to induce the sequence labelers and then apply them to the test corpus.

## Background

Published literature remains the largest resource of scientific information in human society and the explosive growth of these publications poses a significant challenge in information access and processing. In the area of biomedicine this kind of information provides insights into the underlying molecular mechanisms of biological macro-molecular interactions and further pharmacological dynamics. In order to use this information, however, the published literature must be first converted into a structured format suitable for modeling, reasoning, large-scale querying, and further computational analysis. It is a promising and yet challenging task to construct network information involving bio-entities and their events/relationships from biomedical text. Some standards have been established to officially represent biological events like Biological pathway exchange language (BioPAX) [1], the Systems Biology Markup Language (SBML) [2] and the Biological Expression Language (BEL)(http://openbel.org/) [3]. Among them, BEL is gaining increasing attention for system biology applications because it combines the power of a formalized representation language with a relatively simple syntax designed to be both human readable and machine processable.

For assessing the utility of tools for the automated annotation and network expansion, the BioCreative community proposed a challenging task of automatically extracting casual network information in the Biological Expression Language (BEL) format from biomedical literature. BEL is designed to represent scientific findings in the field of life sciences in a format that is not only computable but also easily editable by humans. The findings are captured through causal and correlative relationships between entities in the format of BEL statements. BEL statements convey causal relationships (*increases* and *decreases*) between two BEL terms or among multiple BEL terms. BEL terms are formed using biomedical entities (gene/protein and chemical abundances, biological and pathological processes etc.) together with functions modifying entities (e.g. *deg()* (degradation), *tloc()* (translocation)). A concept of namespace (e.g. CHEBI) and associated identifiers, e.g. *a(CHEBI:‘nitric oxide’*), is adopted to normalize entities in a flexible way.

Unlike the previous relation extraction task [4–7], where a relationship is purely between two entities, the BEL task aims to discover the hierarchical relations between biomedical entities, meaning that the relationship (*increases* or *decreases*) can hold among multiple entities and complex biomedical functions (such as *complex()* or *tloc()*) can also be involved. Taking the following sentence together its corresponding BEL statement extracted from BC-V corpus as an example, it illustrates a hierarchical relation involving three entities, one function and one relationship: the catalysis of the protein IL-2 increases the complex between the protein LYN and the protein IL2RB.

The association of *lyn* with *IL-2Rbeta* was markedly elevated by *IL-2* stimulation. (PMID: 11131153).

cat(p(HGNC:*IL2*)) increases complex(p(HGNC:*LYN*), p(HGNC:*IL2RB*)).

The primary challenge on this task is that a BEL statement is annotated in the BC-V training corpus in a sentence-level fashion, making it difficult to directly apply conventional machine learning approaches. The previous studies, therefore, either adopt rule-based methods [8, 9] or apply event extraction/semantic role labeling models induced from other training corpora [10–13] and then transform these structures to BEL statements. One main drawback of these methods is that the training corpus of the BC-V BEL task, which contains roughly 6 K informative sentences, is essentially unexplored. Ali et al. [14] directly use the BEL training corpus. They induced a CNN model from training corpus but complex relations and biomedical functions are totally ignored, and, therefore, the performance is greatly diminished.

We cast the BEL statement extraction task as a hierarchical sequence labeling problem and generate an instance-level training corpus via word alignment from the BC-V training corpus. The basic idea is first to align a sentence-level BEL statement with its corresponding sentence, i.e., label the text segments with hierarchical tags corresponding to entities, functions and relations respectively in the BEL statement using a word alignment algorithm. Then, hierarchical sequence labeling models are trained from the tagged sentences and apply to the test sentences in order to extract and reconstruct the BEL statements. Our contributions include:

- Generating an instance-level training labeled corpus from the sentence-level training corpus via word alignment technique for the BEL statement extraction task.
- A hierarchical sequence labeling method for extracting causal network information, where the higher layer model is based on the immediately lower one.
- We achieve the F1 performance of 31.6% on the statement level on the BC-V BEL task and promising performance on the BC-VI BEL task.

## Methods

In our approach, the BEL statement extraction task is casted as a hierarchical sequence labeling problem. The basic idea is that the lowest layer deals with the task of Named Entity Recognition (NER), i.e. to recognize bio-entities from the biomedical text, then the second layer identifies functions for bio-entities from the sequence of bio-entities and words, afterwards the upper layers detect relationships based on the bio-entities and functions recognized in the lower layers, and finally BEL statements can be constructed from the recognized bio-entities, functions and relations.

**Fig. 1:**
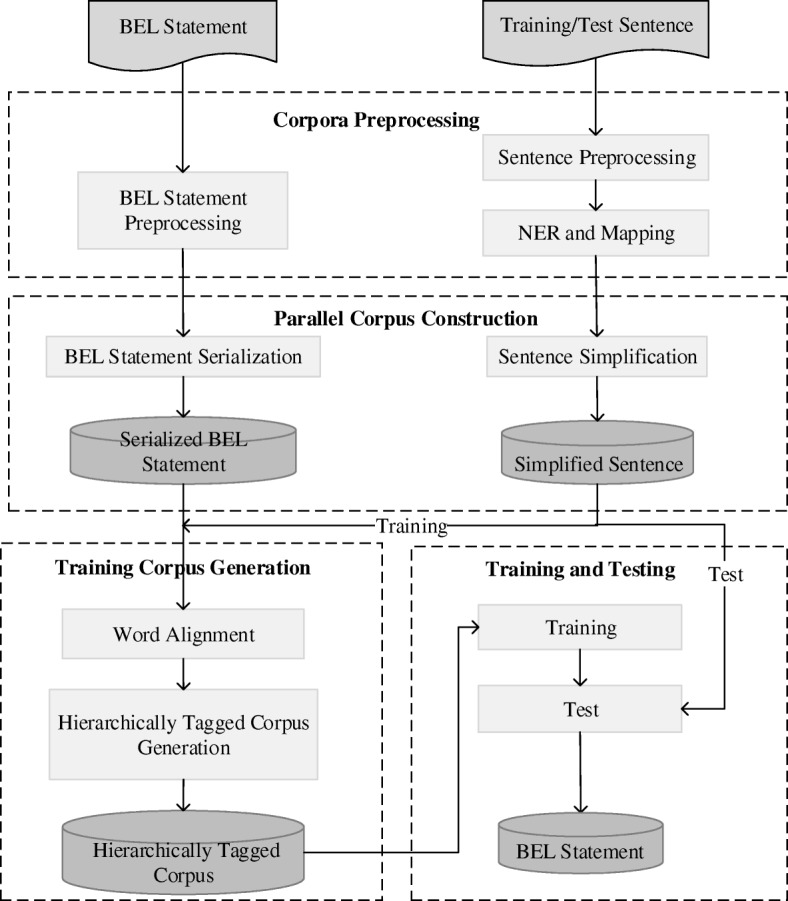
The workflow of our approach to BEL statement extraction

Figure 1 illustrates the framework of our approach. It consists of four major pipelined components: Corpus Preprocessing (CP), including Named Entity Recognition and Alignment (NERA), Parallel Corpus Construction (PCC), Training Corpus Generation (TCG), Model Training/Testing (MTT). During corpus preprocessing, training/test sentences are tokenized and BEL statements are normalized. The NERA module recognizes entities in a sentence and align them with their identifiers in BEL statements. The PCC module constructs a parallel corpus between simplified sentences and the corresponding BEL statements. The TCG model generates training instances by means of applying a word alignment tool to the parallel corpus in order to obtain alignments between words and BEL nodes. Finally, hierarchical sequence labeling models are trained from the training instances and applied to predict on the test sentences and the predicted results are converted to the BEL statements. Note that the training/test sentences experience the same sequence of Sentence Preprocessing, NERA, and sentence simplification etc. except that the training sentences are fed into the Training Corpus Generation while the test sentences are taken as the input to the testing module to predict its hierarchical labels.

**Fig. 2:**
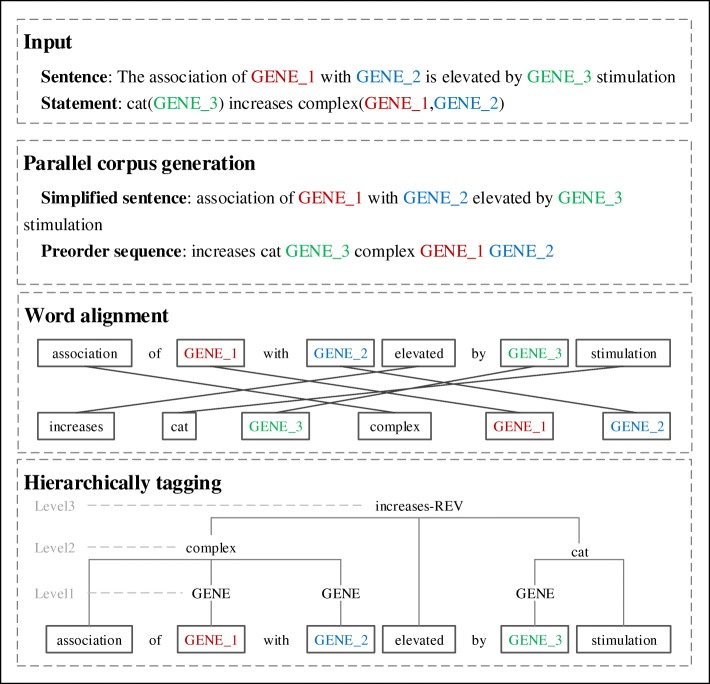
Generating the training corpus for hierarchical sequence labeling via word alignment

As shown in Fig. 1, the construction of a hierarchically tagged training corpus plays a key role in our approach. As an example Fig. 2 illustrates the steps in a top-down manner to generate this instance-level corpus from the BEL training corpus for a specific sentence/statement pair. The input is the original pair from the BC-V training corpus where the biomedical entities have been identified and replaced with their placeholders. First, the sentence is simplified to a word sequence according to its shortest dependency path connecting these entities, and the statement is converted to its preorder node sequence. These statements and sentences are regarded as the respective source and target language sentences in a parallel corpus. Then, a word alignment tool is applied to the parallel corpus to find the alignment between words in the sentence and nodes in the statement. Finally, a hierarchically tagged corpus can be generated based on the alignment between words and nodes.

### Preprocessing

Preprocessing the training corpus entails two steps: sentence tokenization and BEL statement normalization. For sentence tokenization, we use a simple yet effective way. We do not follow the general tokenization procedure in the news domain, instead it is mainly aimed to facilitate the dictionary-based entity search described in the next subsection. The rules for tokenization are as follows:

1. In addition to the intuition that a comma followed by a space usually means the end of the sentence, we perform special processing for the comma followed by a character or a digital as it usually means a part in a biomedical entity.
2. The hyphen in a composite noun will be tokenized if the noun ends with “ed” or “ing”, because the past participles and gerunds included in the noun are usually associated with some kind of relationships in biomedical literature. For example, the hyphen in “progesterone-induced” should be separated to facilitate the subsequent entity search and relation extraction.
3. Some special tokens are separated since they may contain parameters for some BEL functions. For example, “Ser727” is tokenized as “Ser” and “727” because the former is an abbreviation of the amino acid “Serine” and the latter is a base position in the protein. They are potentially useful for function (*pmod()*) recognition.

Meanwhile, we normalize the BEL statements by resolving the redundancy and inconsistency among them, e.g. there are some cases where two identical statements correspond to the same sentence and other cases where the same entities are involved in two distinct BEL statements. Additionally, in order to facilitate the serialization of the BEL statements, we elevate the hierarchical level of some protein modification functions (including *pmod()*, *sub()*, and *trunc()* etc.) within an entity by reorganizing the entity and the parameter of the function as the child nodes of the function itself. For example, the BEL component “p(HGNC:AKT1, pmod(P, S, 21))” is converted to “pmod(p(HGNC:AKT1), P, S, 21)”, thus keeping the function always above the entities in the relation hierarchy.

### Named entity recognition and alignment

Since only the identifiers of entities, rather than their exact locations in a sentence, are given in the corpus, the first step is to recognize biomedical entities in the sentence and to align them to their identifiers in the BEL statement. We adopted three steps including NER, renormalization and dictionary search in order to maximize the entity recall.

#### NER

Three NER tools are used respectively to identify different biomedical entities, including GNormplus [15] for gene and protein recognition, tmChem [16] for chemical recognition and DNorm [17] for disease recognition. In addition, these tools also normalize recognized entities to the corresponding entity databases. GNormplus links genes and proteins to Entrez [18], tmChem links chemicals to MESH [19] and CHEBI [20], and DNorm links diseases to MESH and OMIM [21]. The normalized entities are finally aligned to their identifiers in the BEL statement.

#### Renormalization

Due to name variation, entity identifiers in the BEL statement, however, are not always the same as the ones recognized by the NER tools, so the second step is to renormalize and align the latter into the former. Protein identifiers are consistent across Entrez, HGNC and MGI, so no conversion is needed. Recognized chemical identifiers are converted to CHEBI ones in terms of their normalized names. Recognized disease identifiers are discarded if they are linked to OMIM since conversion from OMIM to MESH is currently infeasible.

#### Dictionary search

Although the three tools achieve the state-of-the-art performance in recognizing different biomedical entities, there are still a number of entities in the BEL statement unrecognized, particular for biological processes. Therefore, we finally performed a dictionary-based entity search for the remaining unaligned entities in the BEL statement. The dictionary consists of symbols and synonyms from five entity lists provided by the organizer, i.e., MGI, HGNC, CHEBI, MESHD and GOBP etc. The matching is based on edit distance and the continuous word sequence with minimal distance to the dictionary entries is recognized as the correct entity and aligned to the BEL statements. For eliminating the variability of entity names and their lengths, we anonymize the entity mentions in sentences by replacing them with placeholders to indicate their types and numbers as GENE_1, GENE_2 in Fig. 2.

### Parallel corpus construction

**Fig. 3:**
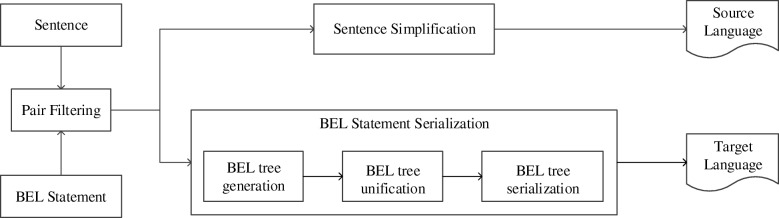
The workflow for parallel corpus Construction

In order to obtain the alignment between the hierarchical relations in the BEL statement and the words in the sentence, a parallel corpus is generated from the sentence/statement pair in the original training corpus where entities in the sentence have been identified and mapped to the BEL statement. Figure 3 presents the generation process, including sentence/statement pair filtering, sentence simplification and statement serialization which is further divided into three steps as BEL tree generation, BEL tree unification and BEL tree serialization.

**Pair Filtering:** When all the entities in BEL statements to a sentence are aligned to the corresponding sentence, the statement/sentence pair is passed to the next step, otherwise it is filtered out.

#### Sentence simplification

**Fig. 4:**
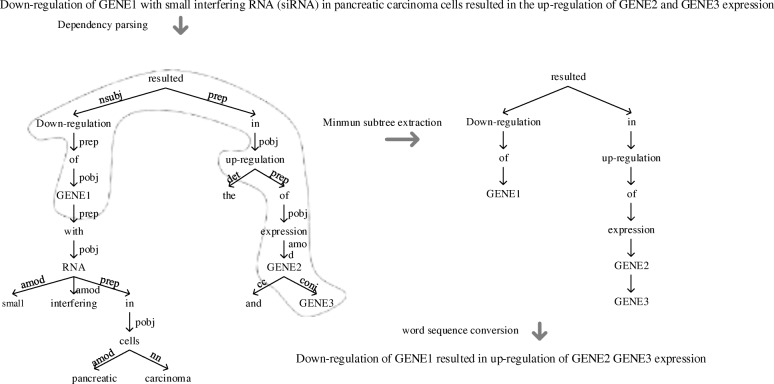
An example for sentence simplification

Essentially the BEL statement can be regarded as a kind of highly condensed semantic representation of the sentence. Direct alignment between the whole sentence and the BEL tree may produce many unaligned words, therefore, a dependency-based simplification method is adopted to simplify the sentence without losing informative words. Stanford parser [22] is used to parse the sentence into a dependency tree and then the words in the minimal subtree, containing all the entities in the BEL statement, are rendered to the simplified sentence according to their original orders in the whole sentence. Figure 4 shows an example sentence “Down-regulation of GENE_1 with small interfering RNA (siRNA) in pancreatic carcinoma cells resulted in the up-regulation of GENE_2 and GENE_3 expression” can be simplified to “Down-regulation of GENE_1 resulted in up-regulation of GENE_2 GENE_3 expression”, which conveys concisely the meaning of the BEL statement.

#### BEL tree generation

**Fig. 5:**
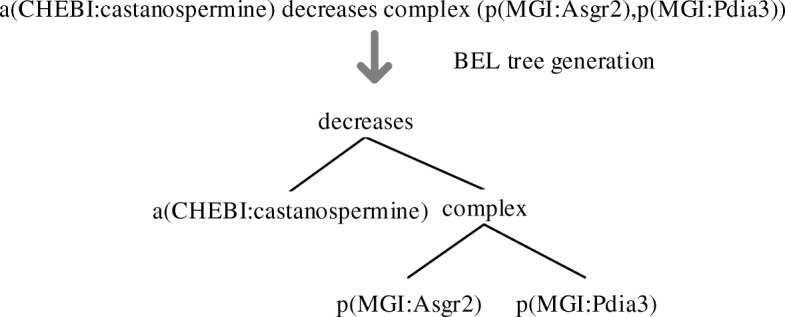
An example for BEL tree generation

In order to serialize BEL statements to get their node sequences, they are first converted into tree structure. The aforementioned preprocessing of BEL statements can ensure the success of this conversion. For a BEL statement, the relation is taken as the tree root, and then the relation’s left/right arguments are converted in their original order into the children of the tree root. This process can be proceeded in a recursive way until a tree is finally generated. Figure 5 shows an example for the process.

#### BEL tree unification

**Fig. 6:**
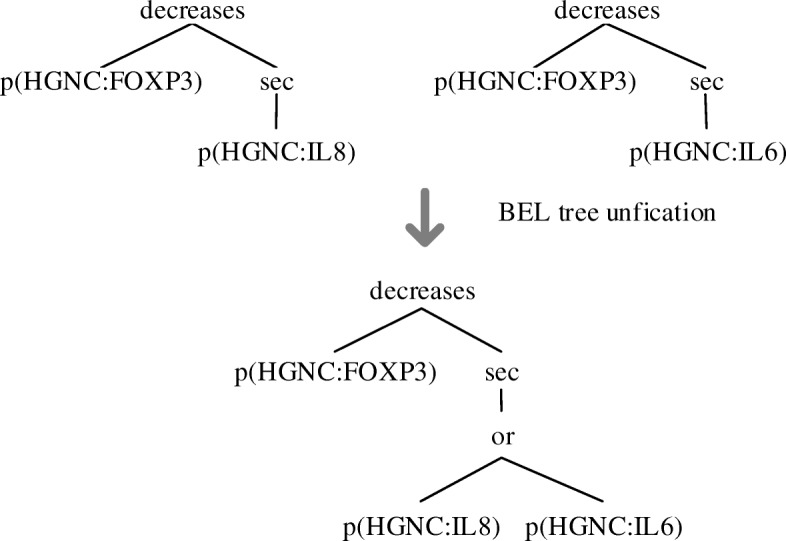
An example for BEL tree unification

One sentence may correspond to multiple BEL trees. Multiple trees with coordination or independent relations can be unified by inserting an additional node “or” to produce a single tree in order to align with the sentence. For example, Fig. 6 shows the process that two BEL trees are merged into a unified tree.

#### BEL tree serialization

With the unified BEL tree at hand, it can be easily transformed into a sequence of nodes via preorder traversal. For example, the above tree “(decreases (a CHEBI:castanospermine) (complex (p MGI:Asgr2) (p MGI:Pdia3)))” is serialized as the node sequence “decreases@2 CHEM_1complex@2 GENE_1 GENE_2” using the serialization method [23], where the sign “@n” following function or relation nodes mean those nodes have *n* children. This number is used to reconstruct the tree structure from the node sequence without ambiguity. Here entity names are replaced with placeholders consisting of entity type name plus the order number of the entity in that type.

### Training Corpus generation

Generating instance-level training corpus from the aforementioned parallel corpus follows two steps: word alignment and hierarchical tag generation.

#### Word alignment

With the simplified sentence as the source language and the serialized BEL statement node sequence as the target language, their alignment can be readily obtained via GIZA++ [24]. The only problem here is that in order to ensure that entities in the sentence be aligned to the same entities in the BEL statement sequence, many pseudo-parallel sentences like “GENE_1 → GENE_1” are augmented to the parallel corpus. For example, the alignment result of the above node sequence and the simplified sentence can be represented as “Preincubation/ with/ CHEM_1/CHEM_1 prevented/decreases@2 association/complex@2 of/ GENE_1/GENE_1 to/ GENE_2/GENE_2”, where in an aligned word pair the left one comes from the sentence and the right one comes from the statement sequence. It occurs that some words in the sentence cannot be aligned to any node in the sequence.

#### Hierarchical tag generation

Based on the alignment result between the nodes in the BEL statement sequence and the words in the sentence, a bottom-up labeling approach is used to annotate the sentence with tags corresponding to BEL nodes layer by layer. The lowest level is for entity and other parameters (such as P, S, or numbers for *pmod*), the immediate upper level (function nodes) is annotated for the text segment spanning between the word aligned to the function node and the words covered by the function node. Finally, the top node (the relation node) is reached and its text span is determined. This process can be schematically illustrated in the last step in Fig. 2.

### Training

Given the hierarchically tagged corpus, we can train hierarchical sequence labelling models using the open source CRF package CRF++ [25] as the fundamental sequence labeler.

**Fig. 7:**
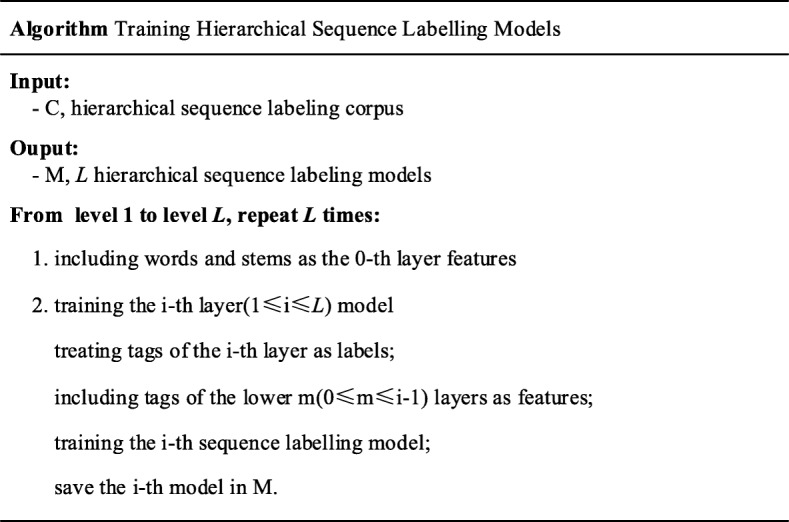
Training algorithm for hierarchical sequence labeling

The training algorithm for hierarchical sequence labeling is described in Fig. 7. If the maximal layer in the training instances is denoted as *L*, then we need to train *L* sequence labeling models. The first layer model is trained using features from words and stems, and then the second layer model is trained using features and labels from the first layer. In this recursive way we can finally obtain the top-layer model.

In every layer of training models, the “BIESO” (begin, in, end, single and out) labeling scheme is used to denote token labels. In traditional sequence labelling-based NER, this scheme usually exhibits the best performance. Note that the features used in k-th layer CRF model include context words and labels in all the lower k-1 layers with window size 5 around the current word.

### Testing

The testing phase includes two steps: model testing and BEL statement construction. The first step uses the *L* models trained above to label the test examples in the same order as when we train them. Differently from training, when labeling the k-th layer, the labels automatically recognized in the lower k-1 layers are treated as features. After labeling all the layers, the second step converts the labeling results into BEL statements. This process is basically the reverse one of training example generation and can be divided into three sub-steps:

#### BEL tree generation

Convert the hierarchical labelling result of the test sentence to the BEL tree structure.

#### Unified tree splitting

If there is “or” nodes in the tree, separate the tree into multiple subtrees accordingly.

#### BEL statement generation

Convert every tree into a BEL statement, including normalizing entity type names and moving some modification functions (*pmod*, *sub* and *trunc* etc.) into the entities.

## Results

### Corpus and evaluation metrics

The original training corpus for the BC-V BEL task contains 11,066 statements and 6353 sentences. After preprocessing and NERA, 4352 statement/sentence pairs are generated as the parallel corpus. After word alignment, 2900 statement/sentence pairs are successfully obtained as the instance-level training corpus for hierarchical sequence labeling. Evaluation in State 2, where the entity identifiers involving in BEL statements are given, is performed on the BC-V test dataset, which is composed of 105 sentences and 202 statements.

The performance is measured in terms of standard P/R/F1, however, due to the complexity of BEL statement extraction, different scores are also calculated in order to evaluate the performance on different extraction levels, i.e. Term, Function-Secondary, Relation-Secondary, Relation and Statement [26]. In particular, evaluation scheme does not discern between direct and indirect relationship types, which means that *increases* and *directlyIncreases* are treated as equal, so are *decreases* and *directlyDecreases*, and function evaluation is simplified by mapping activity functions, such as *kin()*, *tscript()*, and *cat()*, to the more general *act()* function [27]. Among evaluation levels, the statement one is the overall performance that we are most concerned with.

### Experimental results

#### Performance in stage 2 on the BC-V test set

Table 1 reports the performance in Stage 2 on various levels on the BC-V test set with gold entities. From the table we can see that the overall statement F1 is slightly more than 30%, suggesting that in general the BEL statement extraction is a challenging task which deserves intensive research. The table also shows:

1. The performance on Term level is the highest with over 90% of F1, due to nearly perfect precision (~ 100%) and relatively low recall (over 80%). One reason for such performance is that in Stage 2 all the entities participating in BEL statements are given, leading to high precision. Secondly, the high recall (~ 80%) on Relation-Secondary indicates that most relations are recognized, as a result most entities are involved in the final BEL statements.
2. The performance experiences a drastic decline from Relation-Secondary level (88% of F1) to Relation level (~ 44% of F1). The main reason is the stricter evaluation criterion on Relation level, i.e. all three arguments in a relation, including relation types and the argument order, are evaluated. On the other hand, the relationship, which is often conveyed by some key words in the sentence, can be identified much better than the relation tuple with its two arguments.
3. The performance on Function-Secondary/Function level is quite low (~ 30%), particularly in recall (~ 20%). This means that the function recognition is a challenging subtask. The reason maybe that, different from relation extraction where the segment between two involved entities mainly conveys the relationship, it is hard to tell which part of the entity context expresses its function. Meanwhile, additional background knowledge is sometimes needed in addition to the entity context in order to identify its function.
4. The performance on Statement level is drastically lower than that on Relation level. The reason is that there are more than 1/3 BEL statements containing functions and complex relations, and the low performance (particularly the lower recall) on Function level significantly implicates the performance on Statement level.

**Table 1:** Performance in Stage 2 on the BC-V test set

Evaluation Levels	P(%)	R(%)	F1(%)
Term	99.6	83.7	90.9
Function-Secondary	61.1	21.2	31.4
Function	52.4	18.0	26.8
Relation-Secondary	97.6	80.2	88.0
Relation	51.7	38.1	43.9
Statement	37.7	27.2	31.6

#### Performance in stage 2 on the BC-VI test set

Table 2 shows the performance in Stage 2 on various levels on the BC-VI test set with gold entities. Since the gold BEL statements of the BC-VI test set is not public now, the statistics of the test set is unavailable and the evaluation scores are provided by the task organizer.

**Table 2:** Performance in Stage 2 on the BC-VI test set

Evaluation Level	P(%)	R(%)	F1(%)
Term	98.8	83.0	90.2
Function-Secondary	58.8	13.3	21.7
Function	38.9	7.4	12.4
Relation-Secondary	96.6	74.7	84.2
Relation	52.9	35.5	42.5
Statement	32.0	17.5	22.7

We can see in Table 2 that the performance on various levels follows the similar trend to those on the BC-V test set except that the overall performance decreases significantly. Specifically, the performance on Term level is similar to that on the BC-V test set and the performance on Relation-Secondary/Relation levels is slightly lower than that on the BC-V test set (~ 4%/~ 1.5%). The performance on Function-Secondary/Function is significantly lower than that on the BC-V test set (~ 10%/~ 14%), which is the major reason why the overall performance decreases ~ 9% of F1 compared to that on the BC-V test set.

#### Performance comparison with other systems

In Table 3 and Table 4, we compare our work with the other systems on the BC-V and BC-VI test sets respectively. The tables only report F1-scores on various levels, where the best performance on each level (column) is displayed in boldface. In Table 3 our system achieves the promising overall statement performance with 31.6% of F1, and the best performance on both Term and Relation-Secondary levels. In Table 4, however, our system achieves the overall performance comparable to the other work [14] which used a neural network model induced from the BC-V training corpus. The rule-based one [9] achieves the best performance. Regarding the fact that our system is based on the original training corpus, it still has room for improvement if we use the more advanced machine learning methods and more suitable instance representation.

**Table 3:** Performance comparison with related work in Stage 2 on the BC-V test set

System	Term(%)	Func-Sec(%)	Func(%)	Re1-Sec(%)	Rel(%)	F1(%)
Rule-based [8]	82.4	**56.5**	**30.0**	82.4	**65.1**	25.6
Event-based [10]	54.3	26.1	20.8	61.5	43.7	35.2
NCU-IISR [12]	–	–	–	–	–	33.1
Ours	**90.9**	31.4	26.8	**88.0**	43.9	31.6

**Table 4:** Performance comparison with related works in Stage 2 on the BC-VI test set

System	Term(%)	Func-Sec(%)	Func(%)	Re1-Sec(%)	Rel(%)	F1(%)
Rule-based [9]	86.4	**58.9**	**52.6**	**91.9**	**66.8**	49.6
Event-based [13]	85.5	50.0	39.2	83.6	57.6	31.8
NN [14]	83.4	–	–	83.4	42.5	24.1
Ours	**90.2**	21.7	12.4	84.2	42.5	22.7

## Discussion

Generally, we obtain the F1-scores of 31.6% on the BC-V test set and 22.7% on the BC-VI test set. The low performance, particularly low recall, is mainly caused by the cascaded errors induced during different stages:

- **NER in training.** NERA from the training/test sentences is far from satisfaction, even though the gold entities are given in Stage 2 evaluation, particularly for the biological processes which cannot even be called entities in a strict sense and can only be recognized by string match. Matching these processes from a BEL statement into its corresponding sentence seems infeasible in some cases. For example, the entity ‘GOBP*: “hyperosmotic response”’* in a BEL statement should correspond to the text fragment *‘in response to sorbitol-induced hyperosmolarity.’* in the sentence, which is hard to fulfill due to their significant difference.
- **Dependency parsing.** Although we retrained Stanford parser using the GENIA corpus specifically annotated for biomedical domain, there is still a lot of errors for long sentences in biomedical literature, particularly for coordination conjunctions and PP attachment.
- **BEL tree unification.** When we want to unify multiple trees corresponding to a single sentence in the training corpus, we only consider the coordination and independent relations among trees while ignoring other relations, leading to a deficient instance-level corpus. For example, two BEL statements ‘p(HGNC:*BMP2*) decreases bp(GOBP:“*cell cycle*”)’ and ‘p(HGNC:*BMP2*) increases bp(GOBP:“*apoptotic process*”)’ (PMID:10979940) have the same left entity but different roots and right entities, so they can’t be unified into a well-formed tree. This will reduce the number of the training examples by ~ 20%.
- **Word alignment.** While we finally generate 4352 parallel sentences for word alignment, this scale is evidently insufficient for better alignment compared with millions of parallel sentences in machine translation. The alignment error is mainly manifested in the fact that some nodes are not aligned. For example, the simplified word sequence ‘Both GENE_1 and GENE_2 are activated during BP_1’ with the corresponding node sequence ‘increases@2 BP_1 cat@1 OR@2 GENE_2 GENE_1’, the alignment algorithm does not find an aligned word for ‘cat’ node.
- **Hierarchical sequence labeling.** It is always the case that the machine learning performance depends on the number of the training examples. Nevertheless, there are only 2900 ultimate training instances for hierarchical sequence labeling, leading to the decrease in the labeling performance.

## Conclusion

This paper proposes a framework for hierarchical relation extraction using hierarchical sequence labeling on the instance-level training corpus derived from the original sentence-level corpus via word alignment. Its main advantage is that we can make full use of the original training corpus to induce the sequence labelers and then apply them to the test corpus. There are a number of ways to enhance our extraction system in the future, e.g., adopt alternative learning methods for top-layer relation extraction, improve the NER module to recall more entities in the training/test corpus, adjust the BEL tree unification strategy to include more training examples and augment the parallel corpus from other resources etc.

## Abbreviations

BC-V: BioCreative-V
BC-VI: BioCreative-VI
BEL: Biological Expression Language
CHEBI: Chemical Entities of Biological Interest
CNN: Convolutional Neural Networks
GOBP: Gene Ontology names for Biological Process
HGNC: HUGO Gene Nomenclature Committee
MESH: Medical Subject Headings
MESHD: Medical Subject Headings from the Diseases
MGI: Mouse Genome Informatics
OMIM: Online Mendelian Inheritance in Man
